# Monitoring of alien mosquitoes of the genus *Aedes* (Diptera: Culicidae) in Austria

**DOI:** 10.1007/s00436-019-06287-w

**Published:** 2019-03-16

**Authors:** Ellen Schoener, Carina Zittra, Stefan Weiss, Gernot Walder, Bita Shahi Barogh, Stefanie Weiler, Hans-Peter Fuehrer

**Affiliations:** 1 Institute of Parasitology, Department of Pathobiology, University of Veterinary Medicine Vienna, Veterinaerplatz 1, 1210 Vienna, Austria; 2 Burgauberg-Neudauberg, Austria; 3 Division of Hygiene and Medical Microbiology, Medical University of Innsbruck, Innsbruck, Austria; 4 Dr. Gernot Walder GmbH, Außervillgraten, Austria

**Keywords:** Alien mosquitoes, *Aedes albopictus*, *Aedes japonicus*, Monitoring, Ovitraps

## Abstract

Systematic, continuous mosquito surveillance is considered the most reliable tool to predict the spread and establishment of alien mosquito species such as the Asian tiger mosquito (*Aedes albopictus*), Japanese bush mosquito (*Aedes japonicus*), and the transmission risk of mosquito-borne arboviruses to humans. Only single individuals of *Ae. albopictus* have been found in Austria so far. However, it is likely that the species will be able to establish populations in the future due to global trade and traffic as well as increasing temperatures in the course of global climate change. In summer 2017, a project surveilling the oviposition of newly introduced *Aedes* mosquitoes, using ovitraps, was set up by means of citizen scientists and researchers and was performed in six federal provinces of Austria—Tyrol, Carinthia, Vienna, Lower Austria, Styria, and Burgenland. Eggs of *Ae. albopictus* were identified in Tyrol during the months August and September, while *Ae. japonicus* was found in Lower Austria, Styria, and Burgenland. In Vienna and Carinthia, all ovitraps were negative for *Aedes* eggs; however, *Ae. japonicus* was found for the first time in Vienna in July 2017 during routine sampling of adult mosquitoes. With this project, we demonstrated the benefits of citizen scientists for ovitrap-based mosquito surveillance. The finding of *Ae. albopictus* eggs in Northern Tyrol is not yet a proof of the establishment of a self-sustaining population, although it indicates the ongoing introduction of this species along main traffic routes from Italy, where this mosquito is well established. The risk of establishment of the tiger mosquito in the Lower Inn Valley is therefore a given and informing the public about preventive measures to hinder and delay this development is highly recommended.

## Introduction

Fourty-nine mosquito species (Diptera: Culicidae) belonging to eight genera (*Aedes*, *Anopheles*, *Culex*, *Coquillettidia*, *Culiseta*, *Ochlerotatus*, *Orthopodomyia*, and *Uranotaenia*) have been specified in Austria so far (cf. Zittra et al. 2017a). The establishment of the potential invasive species *Aedes* (*Stegomyia*) *albopictus* (Skuse, 1984) (Asian tiger mosquito) and *Aedes* (*Hulecoeteomyia*) *japonicus* (Theobald, 1901) (Asian rock pool or Asian bush mosquito) in Austria has been in dispute until recently (Seidel et al. 2012; Zittra et al. 2015). Moreover, three additional non-native species, *Anopheles* (*Anopheles*) *hyrcanus* (Pallas, 1771), *Orthopodomyia pulcripalpis* (Rondani, 1872), and *Culiseta* (*Allotheobaldia*) *longiareolata* (Macquart, 1838), have been reported in recent years (Lebl et al. 2013; Seidel et al. 2013a, b; Zittra et al. 2017b, 2015, 2014).

The following three alien mosquitoes are expanding their range in Europe: *Aedes albopictus*, *Aedes* (*Hulecoeteomyia*) *koreicus* (Edwards, 1917), and *Aedes japonicus*. These species originate from Asia and have increased their range in Europe over the past decade (Medlock et al. 2012; Walder et al. 2013; Medlock et al. 2015; Cunze et al. 2016a, b). *Aedes japonicus* originates from a temperate climate zone and while its range is likely to expand into Northern and temperate regions of Europe, it is not able to adapt to warmer climates (Cunze et al. 2016a, b), whereas *Ae. albopictus* is currently restricted to Southern Europe but is expected to expand its range as climate change continues to occur (Cunze et al. 2016a, b). This is of importance, because the Asian tiger mosquito *Ae. albopictus* is known for its high ecological and climatic adaptability and is now found as an invasive species on all continents except Antarctica (Bonizzoni et al. 2013). It is adaptable enough to adjust to more temperate climates (Goubert et al. 2017) and since its introduction into Germany, it has already been able to reproduce in high numbers at certain sites in 2015 (Becker et al. 2017). The Asian tiger mosquito is of public health concern since it is a vector of different arboviruses (Paupy et al. 2009; Schaffner et al. 2013) like dengue (Rezza 2012, Ferreira-de-Lima, and Lima-Camara 2018) and Chikungunya virus (Poletti et al. 2011; Carrieri et al. 2012). While *Ae. japonicus* appears to be of lesser public health importance, it is a potential vector for West Nile virus at least in laboratory experiments (Wagner et al. 2018). In Austria, *Ae. japonicus* was firstly observed in 2011 in Styria near the Slovenian border in an artificial water container (Seidel et al. 2012). Between 2010 and 2015, it is assumed that the species expanded eastwards into Burgenland and westwards into Carinthia (Seidel et al. 2016a, b). Since then, the Carinthian population has possibly spread southwards to Northern Italy. Between 2014 and 2016, findings of *Ae. japonicus* females in Burgenland and Lower Austria (Zittra et al. 2017a) highlight the expanding distribution of this alien species in Austria. Moreover, *Ae. japonicus* was also found in 2015 in Vorarlberg (Western Austria) possibly originating from established populations in Switzerland (Seidel et al. 2016a, b). In contrast, *Ae. albopictus* was not widely distributed as *Ae. japonicus*. Contrarily, *Aedes albopictus* was found once in Austria in Jennersdorf, Burgenland, in its immature stages and as a single female (Seidel et al. 2012) and seemed to be restricted to finding single individuals and clutches in the Inntal valley along transit routes until now as demonstrated by a continuous monitoring of this area using so-called “ovitraps” (Walder 2017).

Ovitraps, which operate by attracting female mosquitoes, have been frequently used in Southern Europe for the surveillance of invasive mosquito species (Carrieri et al. 2012; Velo et al. 2016; Baldacchino et al. 2017; Di Luca et al. 2017; Manica et al. 2017) and are also recommended by the ECDC (European Centre for Disease Prevention and Control 2012). Citizen scientists, interested members of the public participating in scientific studies, have also recently been included for mosquito surveillance in both Germany (Walter and Kampen 2017), Spain (Palmer et al. 2017), and the USA (Jordan et al. 2017). These studies, however, relied on the report of adult female invasive mosquitoes by more or less trained members of the public, while in our own study, we provided ovitraps to all participating volunteers. The use of citizen scientists provides advantages such as increased reach of the study area as well as saving money and time.

In the summer 2017, a surveillance project using ovitraps, which were set up in five federal states of Austria, was performed, to predict and determine the presence and distribution of the alien mosquito species *Ae. albopictus*, *Ae*. *koreicus*, and *Ae. japonicus*.

## Materials and methods

In summer 2017, surveillance of mosquito oviposition, using ovitraps, was performed in six federal states of Austria—Tyrol, Carinthia, Vienna, Lower Austria, Styria, and Burgenland. For this, 70 sampling sites were used in Northern and Eastern Tyrol, 14 in Carinthia, 10 in Vienna, 10 in Lower Austria, 18 in Styria, and 7 in Burgenland. The sampling sites were at ground level in urban and rural gardens and public parks, as well as service areas along the highways in Tyrol. The sampling started at the beginning of June in Tyrol and Carinthia and in July in Vienna and Lower Austria and in August at the remaining sites in Styria and Burgenland. It ended at all sites at the end of October. The traps in Tyrol and Carinthia were sampled weekly, whereas the other sites were sampled once a month for a week. This study included the participation of citizen scientists at the sampling sites in Lower Austria, Styria, and Burgenland. The sampling sites in Vienna were the same as the sites for the routine mosquito surveillance for the city of Vienna, where adult female mosquitoes are collected in CO_2_-baited BG sentinel traps (Biogents, Regensburg, Germany) once per month over a 24-h period in the months May to September.

**Fig. 1:**
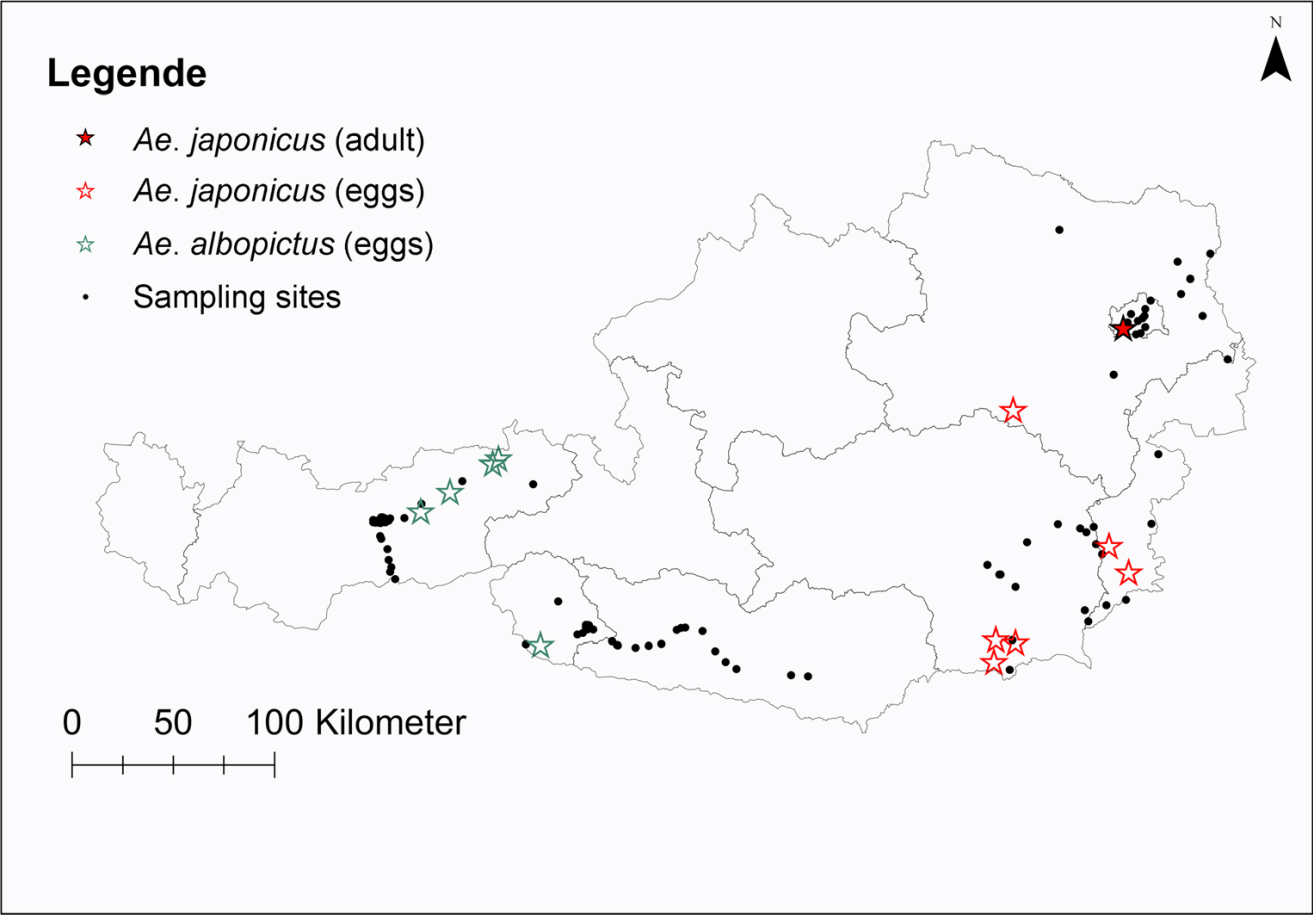
Ovitrap sampling sites for *Aedes* mosquito eggs in six federal states of Austria (Tyrol, Carinthia, Vienna, Lower Austria, Styria, and Burgenland) during summer 2018

The method of using ovitraps has been utilized widely for surveillance of invasive *Aedes* species (Manica et al. 2017) (Fig. 1). We set up two 500-ml black conical cubs per site for 1 week, filled with approximately 400-ml tap water, with wooden paddles (2 × 20 cm—rough plywood) inserted into the water as a substrate for oviposition by the mosquito females. Since we partially relied on the help of citizen scientists, two information sheets were handed out with instructions on how to use the ovitraps. Participating citizen scientists sent used wooden paddles to the lab at the University of Veterinary Medicine in Vienna by mail. After collection, the wooden paddles were examined under a dissection microscope for *Aedes* eggs and the number of eggs present was counted. In Tyrol, the eggs were identified to species-level by morphology and validated by re-examination (MALDI-TOF) at the State Laboratory for South Tyrol Leifers. In the provinces Vienna, Lower Austria, Styria and Burgenland, the eggs underwent to DNA extraction and PCR. DNA was extracted from individual eggs using the Qiagen DNeasy Blood and Tissue kit (Qiagen, Germany). To each sample, 180 μl buffer ATL, 20 μl proteinase K and two ceramic beads (Precellys Ceramic Beads, Peqlab Biotechnologie GmbH) were added and homogenized in a TissueLyser II (Qiagen, Germany). The samples were then incubated at 56 °C overnight and processed according to manufacturer’s instructions. To identify mosquito species, a PCR was performed using the primers LCO1490 and HCO2198 as described in Folmer et al. (1994). Reaction products were commercially sequenced at LGC Genomics GmbH, Germany, and resulting sequences compared for similarity to sequences available on the GenBank® database (http://www.ncbi.nlm.nih.gov/BLAST).

## Results and discussion

**Fig. 2:**
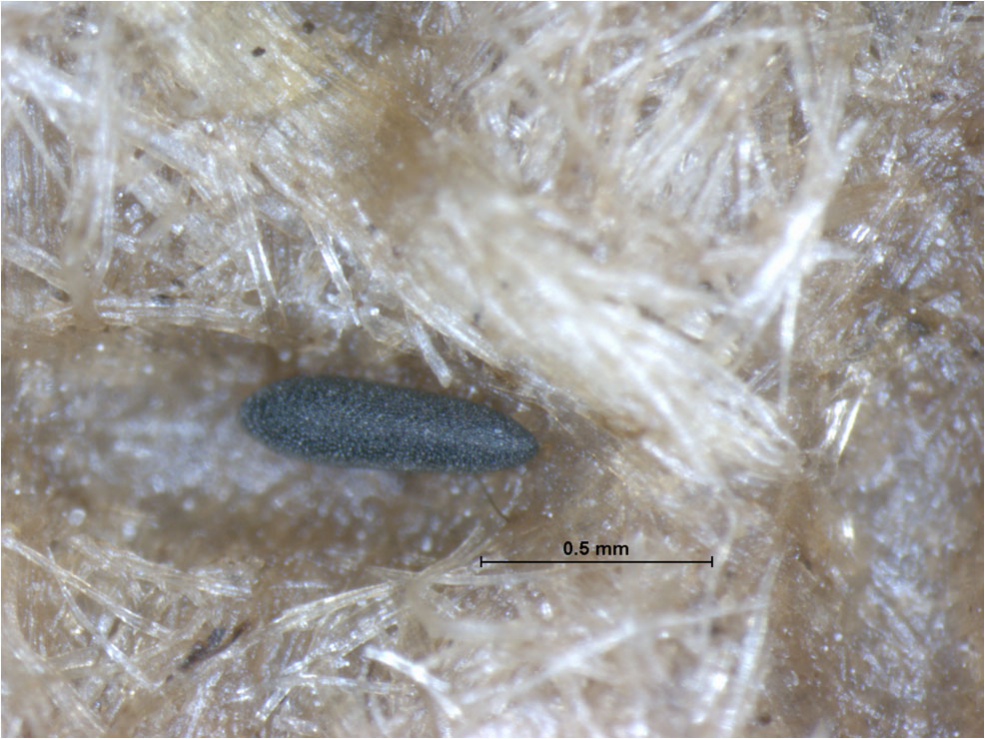
*Aedes japonicus* egg on a wooden paddle from an ovitrap in Lower Austria

During this project, eggs of alien mosquito species were found, but their presence varied by province and month. Eggs of *Ae. albopictus* were only detected in Tyrol along the Inn valley highway, while *Ae. japonicus* was found in Lower Austria, Styria and Burgenland (Table 1). In Vienna and Carinthia, all ovitraps were negative for *Aedes* eggs. However, *Ae. japonicus* was found for the first time during routine sampling of adult mosquitoes in Vienna in July 2017. In Lower Austria, one site was positive for eggs of *Ae. japonicus* in July and August 2017, in Styria, two sites were positive for *Ae. japonicus* eggs in August and one site in September (Fig. 2). *Aedes japonicus* was also identified in Burgenland, where two sites were positive in August. *Aedes albopictus* eggs were only detected in Tyrol, where four sites were positive in August and three sites were positive in September. Most eggs were detected in Northern Tyrol at four different sites. Only one site in Eastern Tyrol was positive for *Ae. albopictus* eggs in August (Table 1). *Aedes koreicus* was not found at any of the examined sites.

**Table 1:** Eggs of alien mosquito species collected in positive individual ovitraps in summer 2017 in the Austrian federal states Tyrol, Lower Austria, Styria, and Burgenland

Month	Calendar week	Province	Town	Species found	Number of eggs present
July	29	Lower Austria	Kernhof	*Aedes japonicus*	9
August	34	Lower Austria	Kernhof	*Aedes japonicus*	12
August	31	Styria	Leibnitz	*Aedes japonicus*	29
August	32	Styria	St.Johann im Saggautal	*Aedes japonicus*	33
August	35	Styria	St. Andrä-Höch	*Aedes japonicus*	52
August	35	Burgenland	Rechnitz	*Aedes japonicus*	12
September	37	Burgenland	Sigleß	*Aedes japonicus*	16
August	34	Tyrol	Tassenbach	*Aedes albopictus*	1
August	33	Tyrol	Weer	*Aedes albopictus*	25
August	34	Tyrol	Weer	*Aedes albopictus*	13
September	36	Tyrol	Weer	*Aedes albopictus*	7
August	35	Tyrol	Münster	*Aedes albopictus*	25
September	36	Tyrol	Langkampfen	*Aedes albopictus*	4
August	34	Tyrol	Kufstein	*Aedes albopictus*	47
September	36	Tyrol	Kufstein	*Aedes albopictus*	52

During this study, 35 citizen scientists participated in Lower Austria, Styria, and Burgenland (Table 2). Participation remained high during the sampling months August to October 2017 (with 91.4% in August and 82.9% in both September and October), although the start of the study was delayed for July due to logistical and financial (delayed start of project funding) reasons. Therefore, only citizen scientists from Lower Austria were participating in July 2017. Drop out reasons for volunteers included holidays abroad, blown over ovitrap cups, loss of wooden paddle, or the sampling date was forgotten.

**Table 2:** Citizen scientist contribution in Lower Austria, Styria, and Burgenland

Month	Participated (out of a total of 35 volunteers): *n* (%)	Number of positive citizen scientist sites (*Ae. japonicus*)
July 2017	8 (22.9%)	1 (12.5%)
August 2017	32 (91.4%)	5 (15.6%)
September 2017	29 (82.9%)	1 (3.4%)
October 2017	29 (82.9%)	0

Eggs of the alien mosquito species *Ae. albopictus* and *Ae. japonicus* were found in four of the examined federal states of Austria during the late summer months of 2017, with *Ae. albopictus* detected in Tyrol only. The Asian tiger mosquito, which is native to South East Asia, is currently restricted mainly to Southern Europe, but due to ongoing climate change is expected to expand its range further northwards (Caminade et al. 2012; Medlock et al. 2015; Cunze et al. 2016a, b). It is established in Italy and has even reached its southernmost Mediterranean islands (Di Luca et al. 2017) and likely played a role in the Italian outbreak of Chikungunya virus in 2007 (Poletti et al. 2011; Carrieri et al. 2012). *Aedes albopictus* is now also found in Switzerland (Flacio et al. 2016) and Germany (Becker et al. 2017), countries neighboring Austria. Most *Ae. albopictus* eggs in this study were found in Northern Tyrol, where the first eggs were documented in 2016 at one site along the main travel routes from Italy (Walder 2017). In 2016, no adult mosquitoes were caught at that particular oviposition site and a singular event was assumed, confirmed in 2017, when no further eggs were detected at the site. However, in contrast to previous years, in 2017, there was repeated evidence of oviposition at several sites with a high egg count in the Lower Inn Valley. Although this does not provided proof for the establishment of a self-sustaining population of the Asian tiger mosquito in Northern Tyrol, the distribution of the positive sites and the time of detection are indicative of continuous introduction and the local reproduction of these introduced individuals. In Eastern Tyrol, *Ae. albopictus* eggs were found for the first time and this shows that tiger mosquitoes were transported northward from Southern Tyrol and Trentino (Italy) and survived long enough to lay eggs.

In future years, it is likely that *Ae. albopictus* will disperse further along travel routes from the South and future surveillance will show if the species is able to establish a self-sustaining population.

Like *Ae. albopictus*, *Ae. japonicus* is expanding its range in Europe (Cunze et al. 2016a, b). During this study, eggs of this species were found in three Austrian states and adult individuals were caught in Vienna for the first time. This finding is not surprising, since *Ae. japonicus* has been spreading in Austria in recent years and has been present in Carinthia, Burgenland, Vorarlberg, and Tyrol (Zittra et al. 2017a; Seidel et al. 2016a, b). It has also been found in the neighboring countries of Germany (Kampen et al. 2016), Hungary (Seidel et al. 2016a, b), and Italy (Seidel et al. 2016a, b).

## Conclusion

With this project, we demonstrated the benefits of citizen scientists for mosquito surveillance using ovitraps. The detection of *Ae. albopictus* eggs in Northern Tyrol is not yet a proof of the establishment of a self-sustaining population, although the distribution of the positive sites and the time of detection are indicative of the ongoing introduction of this species along main traffic routes from Italy, where this mosquito is well established. The risk of establishment of the Asian tiger mosquito in the Lower Inn Valley is therefore high and we recommend informing the general public about preventive measures to hinder and delay this development.
